# A parallel-group randomized clinical trial of individually tailored, multidisciplinary, palliative rehabilitation for patients with newly diagnosed advanced cancer: the Pal-Rehab study protocol

**DOI:** 10.1186/s12885-017-3558-0

**Published:** 2017-08-23

**Authors:** Lise Nottelmann, Mogens Groenvold, Tove Bahn Vejlgaard, Morten Aagaard Petersen, Lars Henrik Jensen

**Affiliations:** 1 Department of Oncology, Palliative Team, Vejle Hospital, Beriderbakken 4, 7100 Vejle, Denmark; 2 Research Unit, Department of Palliative Medicine, Bispebjerg Hospital, Copenhagen University Hospital, Copenhagen, Denmark; 3 Department of Public Health, University of Copenhagen, Copenhagen, Denmark; 4 Danish Colorectal Cancer Center South, Vejle Hospital, Beriderbakken 4, Vejle, Denmark; 5 Institute of Regional Health Research, University of Southern Denmark, Odense, Denmark

**Keywords:** Palliative care, Early integrated care, Rehabilitation, Supportive care, Advanced cancer, Quality of life research, Patient involvement, Randomized clinical trial, Cost-effectiveness, Study protocol

## Abstract

**Background:**

The effect of early palliative care and rehabilitation on the quality of life of patients with advanced cancer has been only sparsely described and needs further investigation. In the present trial we combine elements of early, specialized palliative care with cancer rehabilitation in a 12-week individually tailored, palliative rehabilitation program initiated shortly after a diagnosis of advanced cancer.

**Methods:**

This single center, randomized, controlled trial will include 300 patients with newly diagnosed advanced cancer recruited from the Department of Oncology, Vejle Hospital. The patients are randomized to a specialized palliative rehabilitation intervention integrated in standard oncology care or to standard oncology care alone. The intervention consists of a multidisciplinary group program, individual consultations, or a combination of both. At baseline and after six and 12 weeks the patients will be asked to fill out questionnaires on symptoms, quality of life, and symptoms of depression and anxiety. Among the symptoms and problems assessed, patients are asked to indicate the problem they need help with to the largest extent. The effect of the intervention on this problem is the primary outcome measure of the study. Secondary outcome measures include survival and economic consequences.

**Discussion:**

To our knowledge the Pal-Rehab study is the first randomized, controlled, phase III trial to evaluate individually tailored, palliative rehabilitation in standard oncology care initiated shortly after an advanced cancer diagnosis. The study will contribute with evidence on the effectiveness of implementing early palliative care in standard oncology treatment and hopefully offer new knowledge and future directions as to the content of palliative rehabilitation programs.

**Trial Registration:**

Clinicaltrials.gov Identifier: NCT02332317, registered retrospectively on December 30, 2014. One study participant had been enrolled at the time.

## Background

### Early integrated palliative care

Studies have shown that cancer patients have unmet palliative needs (e.g. physical, social, emotional, and cognitive challenges related to living with cancer) [1].

Palliative care is applicable early in the course of illness and in conjunction with other therapies [2] but is often thought to address “end-of-life”-needs only; perhaps especially in the eyes of professionals involved in oncology, who often play the role of referring the patients to specialized palliative care when all other treatment options are exhausted [3]. Many patients and caregivers associate palliative care with hopelessness and giving up [4]. As a result many patients are not referred to specialized palliative care until late in the disease trajectory [5].

According to The World Health Organization (WHO, 2002) palliative care should not only be considered for the dying but for all patients and families living with a life threatening illness [2] and thus be an integrated part of the treatment at an early stage of the disease. This approach is known as early integrated palliative care. The recommendations are very general and lack specifications about the timing and content of the early, integrated palliative care intervention.

The American Society of Clinical Oncology released a provisional clinical opinion in 2012 about the integration of palliative care into standard oncology care [6] based on seven randomized clinical trials. The conclusion in this expert opinion was that, *while evidence clarifying optimal delivery of palliative care to improve patient outcomes is evolving[…] strategies to optimize concurrent palliative care and standard oncology care, with evaluation of its impact on important patient and caregiver outcomes (*e.g. *quality of life, survival, health care services utilization, and costs and on society, should be an area of intense research.*

This provisional clinical opinion has just been updated reflecting the change in evidence since the previous guideline [7]. The Expert Panel still concludes that more research is needed, especially with inclusion of patients with advanced cancer in early-phase clinical trials.

Several studies have investigated the integration of specialized palliative care into clinical oncology for patients with advanced cancer with promising results. The studies differ, however, in outcome measures, study participants, and timing and contents of the early palliative intervention.

A Canadian study published in 2009 [8] was the first to test the hypothesis that patients with advanced cancer who were offered specialized palliative care shortly after the diagnosis in conjunction with standard care would be more informed and participate more actively in their treatment plan and care. The hypothesis was that this would lead to better quality of life (QoL), better symptom control, lower depression rate and lower health expenses compared with the patients who received standard treatment. A total of 322 patients with newly diagnosed advanced breast, lung, gastrointestinal or genitourinary cancer were randomized. The intervention was a telephone based educational and supportive program led by specialized nurses. The weekly sessions were designed to empower patients to articulate palliative and end-of-life needs to their oncologist. The results showed significantly better self-reported QoL and lower depression rates in the intervention group compared to the control group, whereas no improvement in symptom control or decrease in health expenses was shown.

In an American single center study published in 2010–12 [9–11], 151 patients with newly diagnosed metastatic non-small cell lung cancer were randomized to standard care or standard care plus a palliative care intervention. The intervention was at least one monthly consultation with a specialized palliative care physician and nurse. The primary outcome measure was QoL 12 weeks after randomization. Other outcome measures were anxiety and depression, and health care expenses. The study was not designed to show differences in survival, but the analysis was made post hoc. The result of the study was significantly higher self-reported QoL of the patients who had received the palliative intervention. The intervention group also showed significantly fewer symptoms of anxiety and depression after 12 weeks and even a significantly longer mean survival of 11.6 months vs 8.9 months despite less aggressive active treatment in the intervention group.

The question of timing was evaluated in a large study of early versus delayed initiation of a palliative care intervention published in 2015 [12]. Patients (*N* = 207) with advanced cancer were randomly assigned to receive an in-person specialized palliative care consultation, structured telehealth nurse coaching sessions (once per week for six sessions), and monthly follow-up either early after enrollment or 3 months later. The outcome measures were group differences in QoL, symptom impact, mood, 1-year survival, and resource use. The study showed that the patient-reported outcomes and resource use of early entry participants were not statistically different from that of the late entry participants; however, their 1-year survival was significantly improved compared to those who began 3 months after enrollment.

Other studies of early, integrated, specialized palliative care have pointed to improved QoL [13], better patient and caregiver satisfaction with care, and a high level of satisfaction with the integrated model amongst oncologists due to patient satisfaction, reduction of symptom burden, and time saved in the clinic [14, 15].

### Rehabilitation of patients with advanced cancer

The average survival time of patients with advanced cancer, calculated from the date of diagnosis until death, is improving [16], which makes the discussion of rehabilitation of this group of patients increasingly relevant.

Specialized palliative care is a multidisciplinary approach aiming at relieving suffering in all its dimensions throughout the course of life-threatening disease and for everyone closely affected by the disease [2]. Rehabilitation aims at improving and maintaining physical, mental, social and intellectual performance levels and preventing loss of functions related to activities of daily living (ADL) with the purpose of supporting independence and self-management [16].

The overlap between specialized palliative care and rehabilitation becomes clear when assessing the resources and needs of the individual patient and caregiver, especially early in the disease trajectory. Specialized palliative care and rehabilitation involve many of the same health professionals and can be combined in an ambulatory setting [16].

In Great Britain the “Palliative Day Care Centers/ Services” have existed for decades combining specialized palliative care and rehabilitation. The physical frame is often a hospice. Group activities play a pivotal role with a combination of group discussions of issues related to living with cancer and physical exercise. A review of 15 quantitative and qualitative studies about” Palliative Day Care Centers/Services” conclude that they provide a high degree of patient and caregiver satisfaction and that the possibility of forming relations with the staff and other patients is of great importance [17]. Whether the patients experienced better symptom control or better health related QoL from participating in the services was not clear.

A phase II study from Norway published in 2006 tested the effect of an individually tailored twice-a-week 6-week physical exercise program on physical performance and QoL in patients with incurable cancer and a life expectancy of less than one year [18]. The conclusion was that the exercise program was not only feasible; it also significantly improved the physical and emotional functioning of the patients and reduced fatigue. However, the study population was quite small and did not include a control group.

An American study published in 2015 tested the effect of a multidisciplinary QoL-directed intervention on patients’ adherence to the planned chemoradiation treatment [19]. A cohort of 61 patients with advanced localized gastrointestinal cancer was formed by pooling the results of two randomized, controlled trials using the same intervention. Twenty-nine patients were randomized to participate in sessions of exercise, education, and relaxation two or three times a week for six or eight weeks, and 32 patients received standard medical care. The study found a significantly higher proportion of patients completing the “as planned” cancer treatment and significantly fewer requiring hospitalization in the intervention group compared to the control group, and the results were still statistically and clinically significant when the analyses were completed for the subgroup who received neoadjuvant chemoradiation treatment.

### Aim of this study

The overall experience with early, integrated, specialized palliative care and rehabilitation of advanced cancer patients is positive, but the evidence is still sparse and more research is required.

The Pal-Rehab study combines elements of early, integrated, specialized palliative care with elements of rehabilitation in an individually tailored intervention in patients with advanced cancer.

The aim of this study is to elucidate whether a 12-week individually tailored, palliative rehabilitation program initiated shortly after an advanced cancer diagnosis reduces physical and emotional symptoms/problems and improves QoL. The patient chooses the main symptom/problem to be focused on. Impact on survival and economic consequences measured as health service utilization will also be evaluated.

## Methods/design

### Study design

The study is a phase III, controlled, randomized trial with 300 patients allocated 1:1 to an intervention or a control group at the Department of Oncology, Vejle Hospital. The department offers treatment to adult patients with pulmonary, breast, prostate, colorectal, anal and biliary tract cancer, as well as cancers of the female reproductive organs. On a yearly basis there are around 57,000 outpatient visits and 23,000 radiotherapy fractions and 9300 chemotherapy treatments are administered. Geographically, the patients have up to 1½ hours of transport time by car to the hospital.

**Fig. 1:**
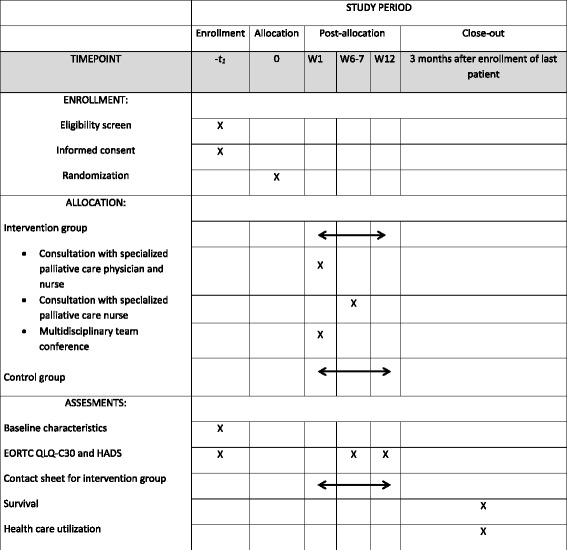
Study outline

Newly diagnosed advanced cancer patients initiating oncologic treatment who consent to participate will be randomized to an individually tailored palliative rehabilitation program alongside their standard oncology care (disease specific treatment) or to standard oncology care alone. Study measures and timepoints can be seen in Fig. 1.

### Study participants

Three hundred patients will be recruited according to the selection criteria (Table 1).

**Table 1:** Selection criteria

Inclusion criteria 1. First-time non-resectable cancer diagnosed less than 8 weeks before enrollment. Patients with prostatic cancer are eligible, if referred to systemic oncologic treatment for the first time less than 8 weeks before enrollment (e.g. due to failure of anti-hormone treatment). 2. Eligible for systemic oncologic treatment at Vejle Hospital and accepts treatment. 3. ≥ 18 years of age. 4. Reads and understands Danish 5. Written and orally informed consent.
Exclusion criteria 1. Other contact with a specialized palliative care unit within 1 year of enrollment. 2. Inability to comply with the protocol due to cognitive or other impairment.

Patients are eligible if the first choice of treatment is systemic and complete surgical removal of the malignant tissue is either ruled out or depends on the success of the systemic treatment. Patients with advanced prostate cancer are often seen at other departments than the Oncology Department following the diagnosis, but they are considered eligible when they are referred to systemic treatment at the Oncology Department for the first time.

### Enrollment procedure and baseline data collection

Eligible patients are informed about the project by a doctor or nurse in the outpatient clinic.

Patients who have signed the informed consent form are asked to complete a baseline questionnaire. Baseline characteristics of the patient (WHO performance status, diagnosis, age, gender, time of primary diagnosis, cancer stage, marital status, and educational background) are registered. Randomization is subsequently performed by the clinical trial unit using a randomization list from randomizer.org [20]. Patients are randomized 1:1 to the intervention or control group with no further stratification used during randomization. The randomization list is blinded from anyone involved in informing potential study participants.

If a patient does not wish to participate, the reason is noted, if possible, and the following characteristics are registered anonymously; diagnosis, age, gender, WHO performance status, cancer stage, marital status and educational background.

### The intervention

The intervention combines elements of specialized palliative care with rehabilitation of cancer patients and is a multidisciplinary assessment of symptoms/problems, QoL, and potential barriers to activities of daily living (ADL) in patients receiving standard oncologic treatment.

The intervention is tailored to the individual patient and since rehabilitation is best described as a process containing specific actions [21], the bundle of actions is investigated rather than single components.

As suggested by Wade [22] the intervention is categorized into five main descriptors; the target population, goals of the intervention, activity or process, resources used, and context (Table 2).

**Table 2:** Description of the individually tailored palliative rehabilitation intervention

Target population	Patients with newly diagnosed non-resectable cancer
Goals of the intervention	Immediate goals • To help patients and caregivers with symptoms/problems identified through questionnaires and a specialized palliative care consultation • To improve overall QoL through symptom control, improvement of physical performance level, and better understanding of the disease and related symptoms Distal/general goals • To improve survival and reduce health service utilization by early recognition of symptoms and problems, improvement of physical performance level and ability to complete “as planned” cancer treatment, and support of patient and caregiver empowerment in future treatment decisions
Activity/process	• Individual consultations • Group educational program for patients and caregivers • Group physical exercise program for patients • Contact to other health departments, the primary sector, local municipality and employer, if relevant and the patient consents
Resources	• Human resources A specialized palliative care team consisting of physicians, nurses, physiotherapists, psychologists, a dietician, an occupational therapist, a social worker, and a hospital chaplain • Physical resources A consultation room, a group room, and a group exercise room. • Time resources The initial specialized palliative care consultation has a duration of approximately one hour plus on average 15 min for the multidisciplinary team conference. The follow-up consultation with a specialized palliative care nurse takes about 30–45 min The group educational program and physical exercise program lasts two and a half hours once a week for twelve weeks. Additional resources are based on individual needs and wishes of patients and caregivers and will be assessed retrospectively
Context	• Scientific context Investigation of early, integrated palliative care and rehabilitation for advanced cancer patients • Organizational structures The specialized palliative care team is organized under the Department of Oncology

Within one week after randomization patients allocated to the intervention are seen in the outpatient clinic by a physician and nurse specialized in palliative care. The themes covered in the first consultation are shown in Table 3.

**Table 3:** Content of the first intervention consultation

• Gaps between wishes for ADL and the patient’s current situation	
• Prognostic awareness	
• Problems with the ”patient/caregiver-role”	
• Sleeping disorders	
• Tiredness and fatigue	
• Problems with memory or concentration	
• Lack of appetite, weight loss	
• Pain, respiratory problems, constipation, and other frequent symptoms	
• Anxiety, worry, sadness	
• Feeling of meaninglessness in the current situation	
• Coping mechanisms of patients and caregivers and potential differences	
• Problems of a socio-economic character or family issues	
• Problems concerning work life	

At the end of the first consultation a plan for the following 12 weeks is made together with the patient and caregivers based on their needs and wishes. The intervention provided reflects the offers of the specialized palliative care team to outpatients, i.e. individual consultations at the hospital, telephone consultations, and/or a palliative rehabilitation group program consisting of weekly group discussions followed by one hour of physical exercise, also in groups. If the group program is relevant to the patient, this will be the main intervention. Caregivers are welcome in the group discussions dealing with a new theme every week for 12 weeks. A dedicated, specialized palliative care nurse guides the group, and other health professionals involved in the group program join in depending on the subject. The headlines of the group discussions reflect the themes covered in the first consultation (Table 3) and patients and caregivers receive an overview, when they join the group. Weekly attendance is not mandatory. A patient participating in the group physical exercise program meets with a specialized palliative care physiotherapist beforehand and an individual exercise program is tailored following a series of tests. When the 12-week program has been completed, the patient is examined by the same physiotherapist and receives tailored advice on future physical activity.

During the 12-week group program the patients and caregivers will have supplementary individual consultations if needed, together or separately. At the end of the program they are offered a final evaluation with the same physician and nurse they met at the first consultation.

Caregivers participating in the group program are encouraged to share their thoughts and experiences with each other in a separate room while the patients do the physical training.

If the patient/caregiver prefers or is better suited for individual consultations instead of the group program, this will be accommodated.

If no further intervention is initiated after the first consultation, the patient and caregivers are provided with contact details and may approach the palliative team any time during the next 12 weeks without a new referral.

All patients and caregivers in the intervention group are discussed at least once at a multidisciplinary team conference. A midway follow-up consultation with a specialized palliative care nurse is held six to seven weeks after randomization.

In order to describe the intervention in detail a “contact sheet” covering all possible elements of the intervention is used for prospective registration. Members of the specialized palliative care team fill in the sheet after each contact with a patient or caregiver. Medical records are also kept for further details.

### The control group

The control group receives standard care at the Department of Oncology. In addition to anticancer treatment all patients have access to a number of paramedical services available through referral (Table 4). These services are not open for caregivers.

**Table 4:** Standard hospital based paramedical care

Nutritional support	• All cancer patients are screened for weight loss at the beginning of each treatment. In case of significant weight loss the patient is referred to a dietician
Physical support	• The patient can be referred to the hospital physiotherapist. The main reason is significant lymphedema • In cooperation between clinical oncology nurses and hospital physiotherapists patients having a high performance level are offered an extensive group based training program 4 times a week for 6 weeks
Phsycosocial support	• The outpatient clinic employs psychologists and social workers, and the hospital has a priest. The mean waiting time for a patient referred to a psychologist is currently 3 weeks

### Outcome measures

#### Study objectives

The primary objective of the study is to assess the impact of the intervention on the symptom/problem prioritized by the patient. Secondary objectives are 1) to assess the impact on the symptoms/problems represented in the questionnaires, including QoL and symptoms of depression and anxiety, 2) to assess the impact on survival, and 3) to analyze economic consequences measured as health service utilization from enrollment until three months after final data collection.

#### Measurement instruments

All participants complete a questionnaire at baseline and six and 12 weeks after randomization. The questionnaire consists of EORTC QLQ-C30, which is a validated and widely used questionnaire for assessing symptoms and QoL in cancer patients [23], and Hospital Anxiety and Depression Scale (HADS), which is also validated and often used for screening and assessment of symptoms of anxiety and depression in palliative care, oncology, and other fields of research [24].

The EORTC QLQ-C30 consists of 30 items in 15 scales. In the present study additional items measuring role functioning, cognitive functioning, social functioning, dyspnea, pain, fatigue, insomnia, appetite loss, nausea/vomiting and constipation were added to the questionnaire to expand these scales to at least four items in each scale. The extra items were taken from the item banks developed for computer-adaptive testing of the EORTC QLQ-C30 dimensions [25–28]. The international clinical validation of the item banks is ongoing.

At baseline the patient prioritizes one “primary problem” from a list of 12 categories of “primary problems” corresponding to 12 of the 15 scales of EORTC QLQ-C30, or “none of the above” (Table 5). At six and 12-weeks follow-up the patient is asked whether he/she has received help with the “primary problem” (yes/no) and if yes, whether the help was “insufficient”, “partly sufficient” or “sufficient”.

**Table 5:** List of possible “primary problems” the study participants to choose from

“Primary problem”	Corresponding outcome measure in EORTC QLQ-C30
Limitations in physical functioning	Physical function scale
Limitations in work and daily activities	Role function scale
Limitations in social life	Social function scale
Problems with memory and concentration	Cognitive function scale
Emotional problems (worry, irritation, depression, tension)	Emotional function scale
Fatigue (tiredness and weakness)	Fatigue scale
Pain	Pain scale
Breathlessness	Dyspnea item
Loss of appetite	Loss of appetite item
Nausea	Nausea and vomiting scale
Constipation	Constipation scale
Trouble sleeping	Insomnia item
None of the above	

The primary outcome measure is the difference between the intervention and control groups in the change from baseline to the weighted average at the six and 12-week follow-up measured as area under the curve (AUC) of the EORTC QLQ-C30 scale/category chosen by the patient (e.g., if pain was designated as ‘primary problem’, the primary outcome is based on the EORTC QLQ-C30 pain scale). If a patient has ticked the box “none of the above” and hence has not chosen any of the 12 scales/categories, the primary outcome measure for this patient will be change in health related QoL corresponding to the “Global health status/QoL”-scale in EORTC QLQ-C30. This way of devising and analyzing an individualized trial outcome has previously been motivated and elaborated, although in the previous study the patients did not select the outcome measure [29, 30]. The expanded EORTC scales (see above) will be used in the primary outcome; the traditional EORTC QLQ-C30 scales will be used in a secondary analysis.

### Statistical analysis and sample size calculation

#### Sample size estimation

Data from other studies using EORTC QLQ-C30 suggest a standard deviation of less than 25 for a difference between the repeated measurements, and a group difference of 10 for clinical relevance [31].

With a risk of type I error of 0.05 and type II error of 0.10, 133 patients are required in each arm. In order to allow for a dropout rate of approximately 10% each arm will enroll 150 patients for a total of 300 patients.

### Plan of analysis

#### Questionnaire data

Analyses will be made using the statistical package STATA, latest version (StataCorp, Texas, USA). The primary analysis will be based on the intention-to-treat principle. If the proportion of lacking answers is higher than 5%, we will use multiple imputations in the primary analysis with the following variables: age, gender, diagnosis, cancer stage, and WHO performance score. The questionnaire data will be transformed according to their respective scoring manuals [32, 24].

Multiple regressions will be applied, since despite the randomized design there may be imbalance between the two arms. We will adjust for “the primary problem” and WHO performance score.

Wilcoxon and Chi2 will be used to investigate whether there are differences between the two groups in relation to the following variables that may be of prognostic importance: gender, age, cancer stage, primary diagnosis, marital status, and educational background. If a significant group difference is found, the respective variable will be included in a sensitivity analysis.

In the analyses we combine the data from the three questionnaires by constructing an AUC using a weighted average of the change from baseline to the 6 and 12-week follow-up. A model for longitudinal data and repeated measurements in the multivariate analysis is applied as a subsequent sensitivity analysis.

Since the primary outcome measure “the primary problem” consists of 13 possible choices, we will also perform separate analyses of patients with the same primary problem.

### Survival

Survival will be described by Kaplan-Meier plots. Patients still alive three months after final data collection will be censored as of that date. A Cox regression model will also be applied controlling for the same variables as in the questionnaire data analysis.

### Economic consequences

Data on the number and length of hospital admissions and treatments, visits to outpatient clinics, emergency rooms, and general practitioners are applied in the evaluation of economic consequences of the intervention. The calculation includes the period from enrolment until three months after final data collection. The data are available from the Danish National Patient Registry [33] and the costs of the different services are obtainable through the Danish Health Authority.

Multivariate analysis is used to compare the two groups controlling for the same variables as in the questionnaire data analysis.

### Other statistical and methodological considerations

A certain degree of cross over and loss to follow-up is expected in this study and will be registered.

The baseline characteristics of responders and non-responders will be compared to elucidate any differences. Likewise, the difference in baseline characteristics of patients who were invited to participate but declined and the study participants will be investigated.

Blinding of study participants and health professionals is not possible. However, the primary data analysis will be conducted in a blinded manner using coded numbers not referring to allocation or identifying patient characteristics.

### Patient and caregiver involvement in the study

Before recruitment was initiated the main intervention of the study (the group program) went through a pilot period where alterations were made based on feedback by patients and caregivers to ensure feasibility and relevance.

The intervention is individually tailored during the 12-week study period. Only the initial and midway consultations are mandatory, which leaves room for a great deal of patient and caregiver involvement in the design of the intervention. The individual primary outcome is also chosen by the patient.

The trial is conducted in close collaboration with the Patient and Relatives Council at Vejle Hospital. The protocol was discussed with and finally approved by the Council, which also has a consulting role during the research process. Dissemination of the study result will also take place in collaboration with the Patient and Relatives Council.

### Ethical considerations

In the study period no effort will be made to prevent patients in the control arm from being referred to specialized palliative care according to the referral criteria of the specialized palliative care team. The Helsinki II declaration is followed unconditionally.

### Time plan

The study is actively recruiting and 68% of the intended study population has been enrolled so far. The analyses are expected to be finalized in 2018.

## Discussion

The Pal-Rehab study is a randomized, controlled, single center, phase III trial evaluating palliative rehabilitation as a supplement to standard oncology care in the hospital setting for patients with newly diagnosed advanced cancer. After baseline assessment the patients will be randomized to either the intervention group receiving palliative rehabilitation involving systematic and tailored elements or to the control group receiving standard care alone. Follow-up measurements allow the detection of group differences in symptom control, mood and QoL. Health service utilization and survival will also be investigated.

To our knowledge this is the first time an individually tailored palliative rehabilitation program including a group offer is being evaluated in a randomized controlled setting where the patient group is not selected by cancer type or performance level. Also, this study seems to be the first to assess whether or not early palliative care is helpful in relation to the primary problem specified by the patient.

### Reflections on the intervention of the study

We want to reach the patients and caregivers soon after time of diagnosis in order to focus on early recognition of symptoms and problems affecting QoL and on empowerment of the patients and caregivers with tailored advice by health professionals.

Patients with advanced cancer have many visits to the hospital, especially during standard oncologic treatment. The rehabilitation program takes place at the hospital where the patient is undergoing oncology care. The hospital setting ensures close collaboration between the specialized palliative care team and other healthcare professionals. Everybody involved in the treatment has access to the same patient record. For instance the physiotherapist can access the patient’s diagnostic imaging reports which is helpful in the individual counselling, e.g. about restrictions due to bone metastases.

The group program is the main intervention. In addition to saving time and resources a group intervention enables patients and caregivers to meet with other people in a similar situation.

With the eligibility criteria of this study the traditional distinction between treatment with palliative and curative intent is avoided. Instead, the target group is defined as patients (and caregivers) living with advanced cancer at the time of initiating oncologic treatment. This definition seems relevant as to the future selection of patients for palliative rehabilitation based on the findings of this study.

### Potential weaknesses and strengths of the study

It is a general problem that the voluntariness of research participation and potential gate keeping may lead to the selection of the most resourceful patients, who may not be the ones most in need of palliative support [34]. This is also true for the present study. Furthermore, patients with severe cognitive impairments are excluded from participation for ethical reasons. The prognostic factors of all patients invited to participate will be registered along with the reasons stated by patients who do not wish to participate. In this way we hope to be able to discuss our findings in the light of the characteristics of the group we have investigated and knowledge about the patients not included.

The complexity of rehabilitation as a process poses great challenges in research and it is difficult to separate the specific features of an intervention from non-specific aspects that nonetheless may have powerful effects [21]. For instance it cannot be ruled out that preexisting beliefs about the effectiveness of the intervention plays a role when patients report their outcome. We have chosen two approaches to this methodological problem; firstly, patients are informed in a neutral manner, where few details about the content of the intervention are revealed before randomization. Secondly, patients are asked to choose a “primary problem” before randomization and are asked at follow-up, whether or not they have been helped with this problem. However, they are not informed that the “primary problem” they have chosen correlates to a scale in the EORTC QLQ-C30 questionnaire and they do not have their baseline questionnaire for comparison when answering the follow-up questionnaires. The primary outcome measurements are based on the scores in EORTC QLQ-C30.

Where the word “rehabilitation” may raise positive expectations, “palliative care” sometimes carries a stigma of death or dying, which will expectedly be a challenge when recruiting patients with newly diagnosed advanced cancer. As part of the oral information the patient and caregivers are specifically asked, if they have any experience with “palliative care” and the meaning of the word. If relevant, they are informed that the aim of the intervention is not “end-of-life support”. Patients are also informed that they can participate in the study regardless of whether they initially feel a need for an intervention or not.

Pal-Rehab is a single center study, which may limit generalization of the results. On the other hand the study only includes healthcare professionals conventionally present at any hospital offering systemic cancer treatment in our country. The physical requirements are one group room and physical exercise facilities. We therefore believe the intervention is transmissible to other departments of oncology.

This study does not specifically evaluate the impact on the caregivers although the intervention does aim to cover the needs of the caregivers if invited by the patient. When evaluating economic consequences, the patient’s utilization of health services is calculated, but expenses such as additional transportation and time off from work in connection with study participation are not considered.

There may be a certain amount of cross-over, since the group offer of palliative rehabilitation for people living with cancer is open through referral outside the protocol. However, according to our experience from the first two years of practice, patients are not referred until rather late in their disease trajectory and substantially later than the patients we are recruiting for this trial.

## Conclusion

The Pal-Rehab study is a randomized controlled trial investigating whether an individually tailored, palliative rehabilitation program initiated shortly after diagnosis of advanced cancer improves the QoL of the patients. To our knowledge this has never been done before. The results will contribute to the evidence on early palliative care in standard oncology treatment and hopefully offer new knowledge and future directions about palliative rehabilitation programs.

## Abbreviations

ADL: Activities of Daily Living
AUC: Area Under the Curve
EORTC QLQ-C30: European Organisation for Research and Treatment of Cancer Quality of Life Core Questionnaire, version 3
HADS: Hospital Anxiety and Depression Scale
QoL: Quality of Life
WHO: The World Health Organization
